# Complications and Outcomes in 39,864 Patients Receiving Standard Care Plus Mechanical Circulatory Support or Standard Care Alone for Infarct-Associated Cardiogenic Shock

**DOI:** 10.3390/jcm13041167

**Published:** 2024-02-19

**Authors:** Jan-Sören Padberg, Jannik Feld, Leonie Padberg, Jeanette Köppe, Lena Makowski, Joachim Gerß, Patrik Dröge, Thomas Ruhnke, Christian Günster, Stefan Andreas Lange, Holger Reinecke

**Affiliations:** 1 Department for Cardiology I: Coronary and Peripheral Vascular Disease, Heart Failure, University Hospital Münster, Albert-Schweitzer-Campus 1, D-48149 Münster, Germany; 2 Institute of Biostatistics and Clinical Research, University of Münster, D-48149 Münster, Germany; 3 AOK Research Institute (WIdO), AOK-Bundesverband, D-10178 Berlin, Germany

**Keywords:** Impella, V-A ECMO, IABP, cardiogenic shock, acute myocardial infarction, mechanical circulatory support, outcome

## Abstract

Background: Temporary mechanical circulatory support devices (tMCS) are increasingly being used in patients with infarct-associated cardiogenic shock (AMICS). Evidence on patient selection, complications and long-term outcomes is lacking. We aim to investigate differences in clinical characteristics, complications and outcomes between patients receiving no tMCS or either intra-aortic balloon pump (IABP), veno-arterial extracorporeal membrane oxygenation (V-A ECMO) or Impella^®^ for AMICS, with a particular focus on long-term outcomes. Methods: Using health claim data from AOK—Die Gesundheitskasse (local health care funds), we retrospectively analysed complications and outcomes of all insured patients with AMICS between 1 January 2010 and 31 December 2017. Results: A total of 39,864 patients were included (IABP 5451; Impella 776; V-A ECMO 833; no tMCS 32,804). In-hospital complications, including renal failure requiring dialysis (50.3% V-A ECMO vs. 30.5% Impella vs. 29.2 IABP vs. 12.1% no tMCS), major bleeding (38.1% vs. 20.9% vs. 18.0% vs. 9.3%) and sepsis (22.5% vs. 15.9% vs. 13.9% vs. 9.3%) were more common in V-A ECMO patients. In a multivariate analysis, the use of both V-A ECMO (HR 1.57, *p* < 0.001) and Impella (HR 1.25, *p* < 0.001) were independently associated with long-term mortality, whereas use of IABP was not (HR 0.89, *p* < 0.001). Kaplan–Meier estimates showed better survival for patients on IABP compared with Impella, V-A ECMO and no-tMCS. Short- and long-term mortality was high across all groups. Conclusions: Our data show noticeably more in-hospital complications in patients on tMCS and higher mortality with V-A ECMO and Impella. The use of both devices is an independent risk factor for mortality, whereas the use of IABP is associated with a survival benefit.

## 1. Introduction

Cardiogenic shock (CS) is defined as a state of critically impaired end-organ perfusion due to a reduced cardiac output. Clinically, it is primarily characterised by hypotension (systolic blood pressure < 90 mmHg), pulmonary congestion and signs of impaired tissue perfusion [1,2,3].

There are conflicting data on the incidence of CS over the past decades, with some authors reporting an increase and others reporting a decrease in incidence [4,5,6]. CS affects approximately 5–15% of all patients with acute myocardial infarction (AMICS) [4,7].

Despite numerous efforts and advances in treatment, AMICS remains associated with significantly increased in-hospital mortality of between 40–80% [4,8,9,10]. 

Treatment of AMICS includes early revascularisation, management of end-organ dysfunction and haemodynamic support with inotropes and vasopressors. Early revascularisation has been shown to be a cornerstone in the management of AMICS, significantly improving mortality [2,11]. However, a substantial number of patients remain in AMICS despite optimal treatment. 

Several temporary mechanical assist devices have been introduced over the past decades. The intra-aortic balloon pump (IABP) has been the most widely used, but recent randomised controlled trials and meta-analyses have failed to show a survival benefit for patients with AMICS undergoing percutaneous coronary intervention (PCI) [12,13]. European guidelines no longer recommend the use of IABP in AMICS in the absence of mechanical complications [14].

Over the past decade, active temporary mechanical circulatory support (tMCS) devices have been increasingly used. The Impella (Abiomed^®^, Danvers, MA, USA) is a continuous axial flow pump positioned retrogradely across the aortic valve. It actively unloads the left ventricle, reducing its end-diastolic wall tension and pulmonary capillary wedge pressure. Depending on the size of the Impella implanted, different blood flow rates can be achieved (2.5 to 5.5 L/min) [15].

Another form of tMCS is the implantation of a veno-arterial extracorporeal membrane oxygenation device (V-A-ECMO). This provides full biventricular support using a centrifugal pump and membrane oxygenator. The drainage and return cannulas are usually placed in the femoral vessels. This results in retrograde aortic blood flow and increased left ventricular afterload, increased myocardial oxygen consumption and increased risk of pulmonary oedema.

The use of both methods has increased significantly in recent years [16]. However, there is a paucity of data on the indication, timing and especially the long-term outcomes of patients treated with these invasive and expensive procedures [17,18]. Most of this information comes from small retrospective studies [18,19]. The lack of robust data leads to a moderate recommendation of class IIB-C by the European Society of Cardiology, which requires careful case-by-case assessment [14].

To better understand the current status quo in clinical practice regarding treatment choice, associated complications and outcomes, we retrospectively analysed a large data set of patients with AMICS from the AOK—Die Gesundheitskasse (local health care funds). 

## 2. Methods

All medical procedures were coded according to the German classification of procedures (OPS), which is the German modification of the International Classification of Procedures in Medicine (ICPM). All applied International Statistical Classification of Diseases and Related Health Problems 10th Revision (ICD-10 GM) and OPS codes used are listed in Supplementary Table S1.

### 2.1. Data Source and Patient Selection 

The AOK—Die Gesundheitskasse consists of 11 regional insurers and is the statutory health insurer for more than 26 million people, covering one third of the German population. A claim of insurance is open to any inhabitant regardless of region, profession, income, age or health status.

We received anonymised data by AOK Research Institute (WIdO, Berlin, Germany) with cardiovascular diseases and selected all patients aged ≥ 18 years, who were hospitalised with a primary diagnosis of acute myocardial infarction and/or cardiogenic shock and use of V-A ECMO or Impella between 2010 and 2017 (index hospitalisation). Baseline characteristics included arterial hypertension, diabetes mellitus, dyslipidaemia, obesity, smoking and other parameters within 2 years prior to index hospitalization. Any previous procedure, such as PCI, CABG surgery or valve replacement was also recorded two years before the index hospitalisation. 

### 2.2. In-Hospital Treatment, Outcome, and Follow-Up

All coded procedures during hospitalisation and diagnoses of shock, death, stroke, bleeding, sepsis and acute kidney injury were considered as in-hospital treatment or outcome. 

### 2.3. Endpoints

The primary endpoint was overall survival. Major adverse events were defined as haemorrhagic/ischemic stroke, in-hospital resuscitation or death. Secondary endpoints were acute renal failure requiring renal replacement therapy, bleeding requiring red blood cell transfusion, sepsis, duration of mechanical ventilation and length of hospital stay.

### 2.4. Statistical Methods

Qualitative data were tested using the two-sided Chi-squared test. Quantitative data were tested with a two-sided Wilcoxon test. 

Overall survival as the primary endpoint was analysed using multivariable Cox-regression models. Models included patient risk profiles at baseline. All 95% confidence intervals (CIs) and *p*-values presented are standard unadjusted and purely descriptive. Hazard ratios (HRs) and unadjusted 95% CIs for all characteristics are shown in the tables and figures.

Overall survival was estimated using Kaplan–Meier estimators for selected time points (1 year, 2 years, 5 years). All analyses were intended to be fully exploratory, i.e., hypothesis generating. No causal conclusions can be drawn, and the data were interpreted accordingly. Statistical analyses were performed using R version 3.6.0 (26 April 2019), R foundation, Vienna, Austria

## 3. Results

A total of 39,864 patients with AMI and CS were enrolled between 2010 and 2017, of which 7060 (17.7%) received tMCS (IABP 5451 (77.2%), Impella 776 (11.0%), and V-A ECMO 833 (11.8%)). Nearly two-thirds of patients were male (61.4%). This proportion was higher in the invasive group (tMCS 68.8% vs. no tMCS 59.8%, *p* < 0.001). Patients receiving tMCS were younger, with the largest difference between the V-A ECMO and the no-tMCS groups (V-A ECMO median 63.7 vs. no tMCS median 75.6 years, *p* < 0.001). Patients receiving any type of circulatory support were more likely to have three-vessel coronary artery disease (tMCS 66.5% vs. no tMCS 47.3%, *p* < 0.001), a history of myocardial infarction (tMCS 33.9% vs. no tMCS 29.4%, *p* < 0.001) and chronic heart failure (tMCS 77.4% vs. no tMCS 70.9%, *p* < 0.001) compared with those treated conservatively. Previous stroke (tMCS 11.8% vs. no tMCS 15.5%, *p* < 0.001) and chronic kidney disease (tMCS 39.5% vs. no tMCS 43.0%, *p* < 0.001) were significantly less common in the invasive groups. Baseline characteristics are shown in Table 1 and Supplementary Table S2.

### 3.1. In-Hospital Treatments and Complications 

Considerable differences were observed between conservatively and invasively treated patients, as well as within subgroups (Table 2). Impella patients were noticeably more likely to also receive PCI (Impella 94.6% vs. V-A ECMO 79.1% vs. IABP 74.7, *p* < 0.001), while V-A ECMO and IABP patients were more likely to undergo coronary artery bypass graft (CABG) surgery (IABP 31.6% vs. V-A ECMO 26.5% vs. Impella 2.32%, *p* < 0.001). 

There were also clear differences between the groups in terms of in-hospital complications. Acute kidney injury requiring dialysis was most common in the V-A ECMO group (V-A ECMO 50.3% vs. IABP 29.2% vs. Impella 30.5% vs. no-tMCS 12.1%, *p* < 0.001). In-hospital resuscitation was performed in 64.5% of patients with V-A ECMO, in 42.2% of patients with IABP, and in 53.6% of Impella patients and 42.0% of no-tMCS patients (*p* < 0.001).

Bleeding complications were more common in the V-A ECMO group compared with IABP and Impella groups (V-A ECMO 38.1% vs. IABP 18.0% vs. Impella 20.9% vs. no-tMCS 12.1%, all *p* < 0.001). This includes major bleeding complications, such as haemorrhagic stroke (V-A ECMO 3.12% vs. IABP 0.97% vs. Impella 1.55% vs. no-tMCS 0.76%, all *p* < 0.001). This was also reflected in the need for packed red blood cell transfusions (V-A ECMO 88.4% vs. IABP 54.2% vs. Impella 51.7% vs. no-tMCS 21.7%, all *p* < 0.001). 

Ischaemic stroke was more common in the V-A ECMO group (V-A ECMO 9.60% vs. IABP 5.30% vs. Impella 3.99% vs. no-tMCS 4.11%, all *p* < 0.001). 

Sepsis was more common in the tMCS group but less so in IABP and Impella subgroups compared with V-A ECMO (V-A ECMO 22.5% vs. IABP 13.9% vs. Impella 15.9% vs. no-tMCS 9.25%, *p* < 0.001). 

### 3.2. Length of Stay and In-Hospital Outcome

Patients on tMCS required mechanical ventilation noticeably more often (tMCS 81.4% vs. no-tMCS 63.9%, *p* < 0.001). The median duration of mechanical ventilation was longest in the V-A ECMO group (V-A ECMO 93 h (IQR: 281) vs. IABP 81 h (IQR: 200) vs. Impella 41.5 h (IQR: 144) vs. no-tMCS 32 h (IQR: 126), *p* < 0.001).

The median length of stay for patients without tMCS was 7 days (IQR: 16). This was noticeably shorter than for patients on V-A ECMO (12 (IQR: 39) days) and IABP (13 (IQR: 24) days, *p* < 0.001). 

### 3.3. Overall Survival

Death during index hospitalization occurred in 59.0% of no-tMCS patients, but in only 50.1% of patients with tMCS (*p* < 0.001). This is mainly due to a significantly lower mortality in the IABP subgroup (47.1%, *p* < 0.001 vs. no tMCS). There was no significant difference in mortality in either the Impella (62.1%, *p* = 0.09 vs. no tMCS) or the V-A ECMO subgroups (58.6%, *p* = 0.79 vs. no tMCS) compared with no tMCS. Furthermore, there was no statistically relevant difference in the comparison of V-A ECMO and Impella (*p* = 0.15). 

Remarkably, mortality during the case chain, i.e., for example in continuing care hospitals or rehabilitation clinics without interim discharge, is subject to considerable changes, especially in the group of V-A ECMO patients. Of the patients who were transferred alive, 7.21% of patients without tMCS, 11.76% of patients with IABP, 11.90% of patients with Impella and 38.26% of patients with V-A ECMO died. This shows a significantly higher mortality within the case chain for patients who required mechanical circulatory support in any form (Table 3 for all relevant *p*-values). 

**Figure 1:**
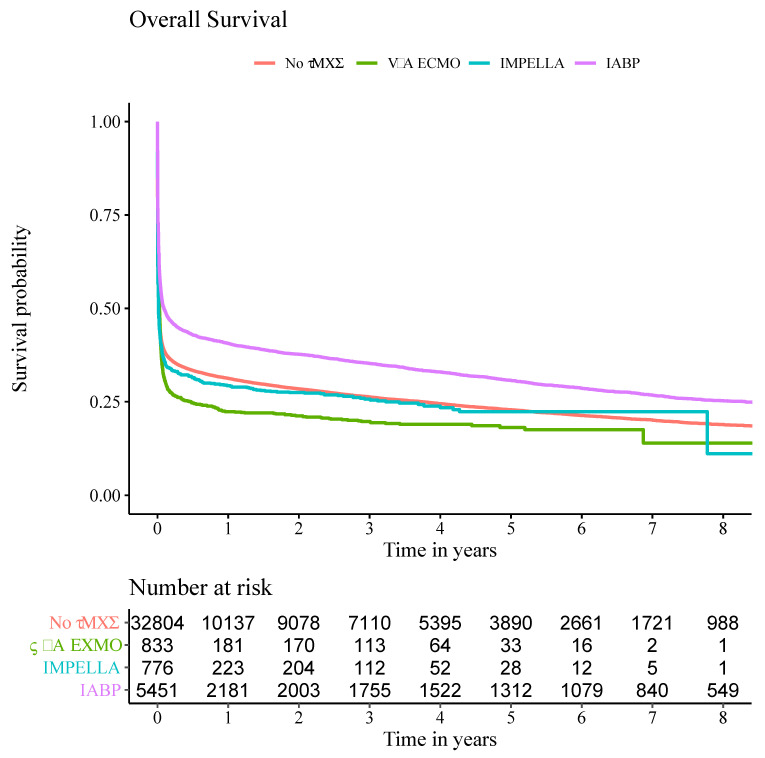
Kaplan–Meier plot for overall survival. Overall survival rates were estimated with Kaplan–Meier estimators for selected time points in patients treated with IABP, Impella, V-A ECMO or without tMCS.

A Kaplan–Meier model for the probability of overall survival is shown in Figure 1. The 1-year Kaplan–Meier survival estimates differed noticeably between groups (no-tMCS 31.3% (95% CI 30.8–31.8%) vs. IABP 40.6% (95% CI 39.3–41.9%) vs. Impella 29.3% (95% CI 26.1–32.5%) vs. V-A ECMO 22.4% (95% CI 19.5–25.2%), all *p* < 0.001). The same was true for 2-year survival estimates (Table 4). The 5-year survival estimates showed consistently similar mortalities for no-tMCS, Impella and V-A ECMO and an increased probability of survival in the IABP group (no-tMCS 22.9% (95% CI 22.4–23.4%) vs. IABP 30.7% (95% CI 29.5–32.0%) vs. Impella 22.4% (95% CI 18.9–25.8%) vs. V-A ECMO 18.1% (95% CI 15.3–21.0%), all *p* < 0.001). 

**Figure 2:**
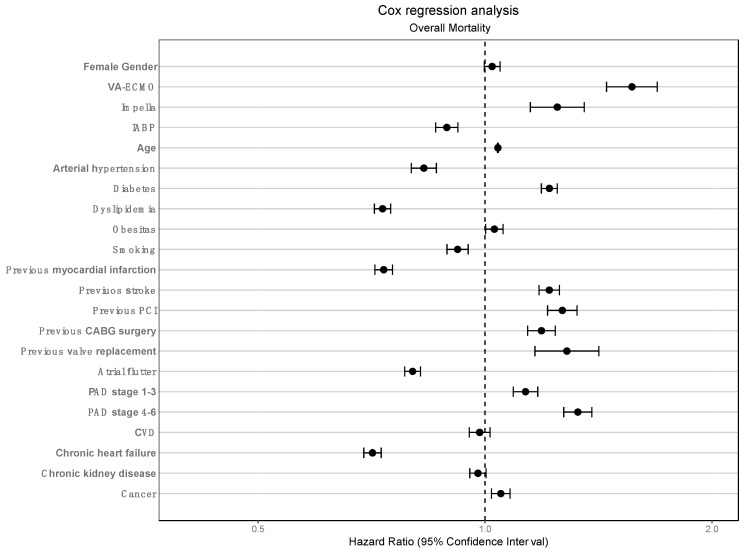
Cox regression model A Cox regression analysis was performed for the adjusted endpoint of overall survival. The use of V-A ECMO and Impella were independently associated with increased mortality, whereas the use of IABP was independently associated with increased survival.

A Cox proportional hazards model was used to adjust for confounders. Classical cardiovascular risk factors, such as arterial hypertension (HR 0.83 (95% CI 0.80–0.86), *p* < 0.001), dyslipidaemia (HR 0.73 (95% CI 0.71–0.75), *p* < 0.001), smoking (HR 0.92 (95% CI 0.89–0.95), *p* < 0.001) or a history of previous myocardial infarction (HR 0.73 (95% CI 0.71–0.75), *p* < 0.001) or chronic heart failure (HR 0.71 (95% CI 0.69–0.73), *p* < 0.001) were associated with improved survival. This also applies to the use of IABP (HR 0.89 (95% CI 0.86–0.92), *p* < 0.001). However, the use of Impella (HR 1.25 (95% CI 1.15–1.35), *p* < 0.001), and especially V-A ECMO (HR 1.57 (95% CI 1.45–1.69), *p* < 0.001), was shown to be an independent risk factor for mortality. Female gender shows at best a trend with regard to a mortality effect (HR 1.02 (95% CI 1.00–1.05), *p* = 0.079). The Cox hazard model is shown in Figure 2 and summarised in Supplementary Table S3.

## 4. Discussion

The gap between the lack of randomised controlled trials investigating the use of tMCS and its increasing use is widening. Limited data are available on the use of V-A ECMO in AMICS, mainly related to its use in IABP-refractory CS [18,19]. To date, there are no convincing data demonstrating relevant advantages of V-A ECMO use in terms of patient-centred outcomes.

Data on the use of Impella are no more conclusive. Most studies have failed to demonstrate a mortality benefit over IABP [18,20]. The use of IABP for AMICS is no longer supported by current guidelines [2] but is still widely used. All of the trials had relatively small numbers of patients. In our analysis, we were able to evaluate the current real-world use of IABP, Impella and V-A ECMO in AMICS in a large, representative cohort from Germany. 

### 4.1. Overall Survival

A key finding is the reported survival rates regardless of treatment choice. In this large database we can show survival rates after AMICS at 1, 2, 5 and 8 years. CS remains a disease with an enormous impact on patients’ short- and long-term survival. Regardless of treatment, the short-term mortality during the index case was a remarkable 57.5% across all groups. Within the case chain, mortality was 61%. It is both remarkable and disturbing that in one of the most advanced healthcare systems in the word, the 5-year survival estimates were only 22.9% (no tMCS), 30.7% (IABP), 22.4% (Impella) and 18.1% (V-A ECMO). This is valuable, up-to-date data that have not been published on this order of magnitude before and it is clear to see that short-term mortality is the main contributor to overall mortality. 

Notably, we did not observe a difference in mortality between patients on Impella or V-A ECMO and those on conservative therapy. Death during the index stay occurred in 58.6% of V-A ECMO patients, in 62.1% of Impella patients and 59.0% of no tMCS patients (Table 3). Interestingly, only 47.1% of IABP patients died during the index hospitalisation This is a very astonishing fact that is also consistent with the Kaplan–Meier estimators. IABP is furthermore the only treatment modality in our data set that was independently associated with improved survival in the multivariate analysis.

A further remarkable peculiarity is seen in the V-A ECMO group. Although the mortality during the index stay was comparable to the mortality in the conservatively treated group, the short-term course within the case chain shows a sharp increase in mortality (58.6% to 74.4%). In other words, 38.3% of the patients who were transferred from the initial hospital after V-A ECMO therapy died in the further course, without having been discharged from an inpatient health care facility in the meantime. The other three treatment groups also showed an expected, but rather smaller increase in mortality during the case chain. The reasons for this are ultimately unclear. It can be assumed that V-A ECMO patients are transferred to further treatment in a markedly worse condition, for example to early neurological rehabilitation, and that treatment goals may be changed, and life-sustaining therapy discontinued, as a result. However, our administrative data do not allow us to provide a concrete answer to this question, so it remains speculative. Nevertheless, multivariate analysis of the data shows that the use of Impella and V-A ECMO are independent risk factors for mortality. A benefit in terms of short- but also longer-term survival cannot be shown.

### 4.2. Treatment Modalities and Complications

**Figure 3:**
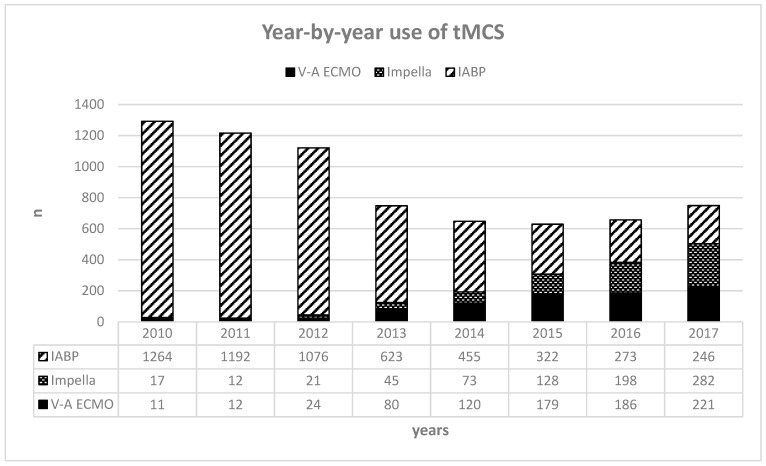
Year-by-year use of tMCS, usage of tMCS devices throughout the query period, and numbers per year. Abbreviations: tMCS—temporary mechanical circulatory support; IABP—intra-aortic balloon pump; V-A ECMO—veno-arterial extracorporeal membrane oxygenation.

First of all, the large number of patients on IABP is astonishing. As the query period covers the years 2010 to 2017, the publication of IABP-SHOCK II [12] was in its first third and a negative recommendation had already been made in 2014 by the ESC/EACTS Guidelines [21]. Although there is a subsequent decrease in IABP use, IABP remains the second most frequently used tMCS device in 2017. A year-by-year overview of the tMCS used is shown in Figure 3.

As expected, due to the invasiveness of the procedures, we found a noticeable age difference between the conservatively and invasively treated patients. This was particularly true in the V-A ECMO group. This could be explained by the non-randomised allocation of these therapies in our cohort. In addition, we observed a trend towards IABP or V-A ECMO in the CABG subgroup, whereas almost all Impella patients were treated with PCI alone. 

The gender differences in all groups were considerable: 61.4% of all enrolled patients with CS were male. Among tMCS patients, the proportion was even more than two thirds (68.8%). This is a striking difference that is consistent across all subgroups. Gender differences in outcomes such as mortality are increasingly recognised and published for a variety of conditions [22,23,24]. The question of whether the lower use of tMCS in women is due to misperceptions on the part of health care professionals and may negatively influence the outcome of female patients must be considered, despite the fact that female gender was not a significant independent risk factor in our data.

There were also important differences in complications during hospitalisation. Bleeding complications occurred most frequently in V-A ECMO (38.1%). The difference between treatment modalities may be due to the required size of the vascular access. Another explanation could be the increased mechanical stress on blood cells, especially platelets, in the extracorporeal circuit and in the centrifugal pump of V-A ECMO. In addition, the slightly more intensive anticoagulation required in V-A ECMO compared with IABP or Impella may play a role, as discussed elsewhere [18]. In view of our survey period and the administrative nature of our data, no conclusions can be drawn about the anticoagulation used and its target values. This is particularly important in view of newer procedures, such as regional anticoagulation on ECMO [25] or the use of bivalirudin [26,27].

Another important point is the different incidence of sepsis between the groups. All types of tMCS were associated with an increased rate of sepsis, which was highest in V-A ECMO group. This could be due to the need for two large vascular accesses as opposed to the single one required for IABP or Impella. Another possible explanation could be the site of the implantation and its urgency. It can be assumed that V-A ECMO implantation under resuscitation conditions in the emergency department is associated with higher infection rates than the more elective implantation of an Impella or IABP in a cardiac catheterisation laboratory under sterile conditions. Unfortunately, our data do not include information on the site of infection, so it is possible that there are other foci of infection apart from device-related infections.

In-hospital resuscitation was significantly more common in the V-A ECMO group compared with the Impella group (*p* < 0.001), and especially so when compared with the IABP and no tMCS groups. This may indicate a more complex and severe disease course in the V-A ECMO group. Another indication is the higher number of patients with renal failure requiring dialysis in the V-A ECMO group. The duration of mechanical ventilation required was also longest in the V-A ECMO group, possibly indicating greater disease severity.

## 5. Limitations

The analysis underlies the general limitations that are linked to a retrospective administrative study design, including missing information on clinical status, parameters (e.g., hemodynamic parameters, laboratory tests, use of catecholamines, shock stage) and time courses, which may be associated with risk of selection bias. It is also not possible to trace the exact time of tMCS implantation and thus gather information about local treatment protocols. Moreover, patients with out-of-hospital cardiac arrest before admission were not included in the data. Subgroup differences can be confounded owing to the lack of propensity score matching. Moreover, due to the retrospective and administrative nature of our data, it must be emphasised that no scoring of disease severity was available and that causality between choice of therapy and mortality cannot be established. It would be very desirable for ICD-10 codes to include data on severity of illness, such as the “Simplified Acute Physiology Score (SAPS) II” [28] or the “Acute Physiology And Chronic Health Evaluation (APACHE) II” score [29].

## 6. Conclusions

We report data that derives from, to our knowledge, the largest cohort of patients with AMICS and who have received various forms of conservative or invasive shock therapy. Our data reflect current clinical practice in the management of these patients in an advanced healthcare system. In addition, our data include long-term follow-up, which shows disappointingly low survival rates after CS, regardless of whether patients were treated invasively or conservatively. Our analysis includes valuable data on in-hospital management and complications. In our data set, the use of V-A ECMO and Impella appears to be associated with increased mortality in the Cox regression model, whereas the use of IABP appears to be associated with decreased mortality. Randomised controlled prospective trials with homogeneous control and intervention groups and clear definitions of cardiogenic shock and its severity are urgently needed to provide the right treatment to the right patient to maximise therapeutic benefit and minimise complications.

## Figures and Tables

**Table 1:** Baseline characteristics.

	No tMCS (*n* = 32,804)	IABP (*n* = 5451)	Impella (*n* = 776)	V-A ECMO (*n* = 833)	*p*-Value
Male, *n* (%)	19,615 (59.8)	3676 (67.4)	541 (69.7)	638 (76.6)	<0.001
Age, median (IQR)	75.64 (17.0)	71.61 (16.8)	70.61 (17.3)	63.68 (16.9)	<0.001
No of diseased coronary vessels: 0, *n* (%)	7308 (22.3)	283 (5.19)	50 (6.44)	63 (7.56)	<0.001
No of diseased coronary vessels: 1, *n* (%)	4056 (12.4)	586 (10.8)	86 (11.1)	72 (8.64)	<0.001
No of diseased coronary vessels: 2, *n* (%)	5922 (18.1)	938 (17.2)	145 (18.7)	145 (17.4)	<0.001
No of diseased coronary vessels: 3, *n* (%)	15,518 (47.3)	3644 (66.9)	495 (63.8)	553 (66.4)	<0.001
Arterial hypertension, *n* (%)	28,907 (88.1)	4680 (85.9)	654 (84.3)	675 (81.0)	<0.001
Diabetes, *n* (%)	17,073 (52.1)	2820 (51.7)	405 (52.2)	425 (51.0)	0.917
Dyslipidemia, *n* (%)	21,893 (66.7)	3866 (70.9)	544 (70.1)	544 (65.3)	<0.001
Obesitas, *n* (%)	8850 (27.0)	1587 (29.1)	225 (29.0)	249 (29.9)	0.002
Smoking, *n* (%)	6522 (19.9)	1223 (22.4)	197 (25.4)	265 (31.8)	<0.001
Previous myocardial infarction, *n* (%)	9653 (29.4)	1883 (34.5)	252 (32.5)	261 (31.3)	<0.001
Previous stroke, *n* (%)	5097 (15.5)	662 (12.1)	92 (11.9)	78 (9.36)	<0.001
Previous PCI, *n* (%)	2356 (7.18)	449 (8.24)	68 (8.76)	76 (9.12)	0.004
Previous CABG, *n* (%)	2728 (8.32)	418 (7.67)	58 (7.47)	50 (6.00)	0.037
Previous valve replacement, *n* (%)	429 (1.31)	40 (0.73)	0 (0)	13 (1.56)	<0.001
Chronic heart failure, *n* (%)	23,244 (70.9)	4187 (76.8)	606 (78.09)	670 (80.4)	<0.001
Chronic kidney disease, *n* (%)	14,090 (43.0)	2187 (40.1)	294 (37.9)	307 (36.9)	<0.001
Chronic kidney disease with dialysis, *n* (%)	6340 (19.3)	873 (16.0)	136 (17.5)	114 (13.7)	<0.001

Abbreviations: tMCS—temporary mechanical circulatory support; IABP—intra-aortic balloon pump; V-A ECMO—veno-arterial extracorporeal membrane oxygenation; IQR—interquartile range; PCI—percutaneous coronary intervention; CABG—coronary artery bypass graft.

**Table 2:** In-hospital treatments and outcomes.

	No tMCS (*n* = 32,804)	IABP (*n* = 5451)	Impella (*n* = 776)	V-A ECMO (*n* = 833)	*p*-Value
PCI, *n* (%)	21,460 (65.4)	4070 (74.7)	734 (94.6)	659 (79.1)	<0.001
CABG, *n* (%)	1519 (4.63)	1723 (31.6)	18 (2.32)	221 (26.5)	<0.001
In-hospital resuscitation, *n* (%)	13,769 (42.0)	2298 (42.2)	416 (53.6)	537 (64.5)	<0.001
Mechanical ventilation, *n* (%)	20,975 (63.9)	4345 (79.7)	648 (83.5)	757 (90.9)	<0.001
Ventilation, median hours (IQR)	32 (126)	81 (200)	41.5 (144)	93 (281)	<0.001
Acute kidney injury, *n* (%)	8835 (26.9)	1732 (31.8)	341 (43.9)	440 (52.8)	<0.001
Renal replacement therapy, *n* (%)	3975 (12.1)	1590 (29.2)	237 (30.5)	419 (50.3)	<0.001
Bleeding, *n* (%)	3058 (9.32)	980 (18.0)	162 (20.9)	317 (38.1)	<0.001
Red blood cell transfusion, *n* (%)	7131 (21.7)	2954 (54.2)	401 (51.7)	736 (88.4)	<0.001
Hemorrhagic stroke, *n* (%)	249 (0.76)	53 (0.97)	12 (1.55)	26 (3.12)	<0.001
Ischemic stroke, *n* (%)	1347 (4.11)	289 (5.30)	31 (3.99)	80 (9.60)	<0.001
Sepsis, *n* (%)	3034 (9.25)	757 (13.9)	123 (15.9)	187 (22.5)	<0.001
Length of stay, median days (IQR)	7 (16)	13 (24)	6 (22)	12 (30)	<0.001
Death (during index case), *n* (%)	19,367 (59.0)	2569 (47.1)	482 (62.1)	488 (58.6)	<0.001
Death (after index case within case chain), *n* (%)	969 (7.21)	339 (11.8)	35 (11.9)	132 (38.3)	<0.001
Total Death (within case chain), *n* (%)	20,336 (62.0)	2908 (53.4)	517 (66.6)	620 (74.4)	<0.001

Abbreviations: tMCS—temporary mechanical circulatory support; IABP—intra-aortic balloon pump; V-A ECMO—veno-arterial extracorporeal membrane oxygenation; PCI—percutaneous coronary intervention; CABG—coronary artery bypass graft; IQR—interquartile range; Index case = stay in the first hospital after admission; case chain = stay in other medical facilities without interim discharge.

**Table 3:** Chi-square *p*-values for death.

Comparison	Death (During Index Case)	Death (After Index Case)	Total Death (Within Case Chain)
No tMCS vs. tMCS	<0.001	<0.001	<0.001
No tMCS vs. IABP	<0.001	<0.001	<0.001
No tMCS vs. Impella	0.09	0.002	0.009
No tMCS vs. V-A ECMO	0.79	<0.001	<0.001
IABP vs. Impella	<0.001	0.94	<0.001
IABP vs. V-A ECMO	<0.001	<0.001	<0.001
Impella vs. V-A ECMO	0.15	<0.001	<0.001

Abbreviations: tMCS—temporary mechanical circulatory support; IABP—intra-aortic balloon pump; V-A ECMO—veno-arterial extracorporeal membrane oxygenation.

**Table 4:** Kaplan–Meier estimators of survival.

	No tMCS	IABP	Impella	V-A ECMO
1 year estimators, % (95% CI)	31.3 (30.8–31.8)	40.6 (39.3–41.9)	29.3 (26.1–32.5)	22.4 (19.5–25.2)
2 years estimators, % (95% CI)	28.4 (28.0–28.9)	37.7 (36.4–39.0)	27.5 (24.3–30.6)	21.3 (18.5–24.0)
5 years estimators, % (95% CI)	22.9 (22.4–23.4)	30.7 (29.5–32.0)	22.4 (18.9–25.8)	18.1 (15.3–21.0)
8 years estimators, % (95% CI)	18.9 (18.4–19.4)	25.3 (24.1–26.6)	11.2 (0–26.8)	14.0 (7.43–20.6)

Abbreviations: tMCS—temporary mechanical circulatory support; IABP—intra-aortic balloon pump; V-A ECMO—veno-arterial extracorporeal membrane oxygenation; CI—confidence interval.

## Data Availability

All data are stored in a central database at the AOK Research Institute (WIdO, Berlin). We received aggregated and anonymised data of all patients meeting the abovementioned inclusion criteria. The authors confirm that the data used in this study cannot be made available in the manuscript, the supplementary files or in a public repository due to German data protection laws (Bundesdatenschutzgesetz, BDSG). In general, access to data of statutory health insurance funds for research purposes is only possible under the conditions defined in the German Social Security Code (SGB V § 287). Requests for access to data can be sent as a formal application, stating the recipient and the purpose of the data transfer, to the competent data protection authority. Access to the data used in this study can only be granted to external parties under the conditions of the cooperation contract of this research project and after written approval by the AOK Research Institute (WIdO, Berlin).

## Supplementary Materials

The following supporting information can be downloaded at: https://www.mdpi.com/article/10.3390/jcm13041167/s1, Table S1: ICD-10-GM Codes/OPS diagnosis and procedure codes for data retrieval; Table S2: Baseline characteristics (pooled); Table S3: Cox regression.

## References

[1] Pepe M., Bortone A.S., Giordano A., Cecere A., Burattini O., Nestola P.L., Patti G., Di Cillo O., Signore N., Forleo C. (2020). Cardiogenic Shock Following Acute Myocardial Infarction: What’s New?. Shock.

[2] Ibanez B., James S., Agewall S., Antunes M.J., Bucciarelli-Ducci C., Bueno H., Caforio A.L.P., Crea F., Goudevenos J.A., Halvorsen S. (2018). 2017 ESC Guidelines for the management of acute myocardial infarction in patients presenting with ST-segment elevation: The Task Force for the management of acute myocardial infarction in patients presenting with ST-segment elevation of the European Society of Cardiology (ESC). Eur. Heart J..

[3] van Diepen S., Katz J.N., Albert N.M., Henry T.D., Jacobs A.K., Kapur N.K., Kilic A., Menon V., Ohman E.M., Sweitzer N.K. (2017). Contemporary Management of Cardiogenic Shock: A Scientific Statement From the American Heart Association. Circulation.

[4] Babaev A., Frederick P.D., Pasta D.J., Every N., Sichrovsky T., Hochman J.S., Investigators N. (2005). Trends in management and outcomes of patients with acute myocardial infarction complicated by cardiogenic shock. JAMA.

[5] Radovanovic D., Nallamothu B.K., Seifert B., Bertel O., Eberli F., Urban P., Pedrazzini G., Rickli H., Stauffer J.C., Windecker S. (2012). Temporal trends in treatment of ST-elevation myocardial infarction among men and women in Switzerland between 1997 and 2011. Eur. Heart J. Acute Cardiovasc. Care.

[6] Jeger R.V., Radovanovic D., Hunziker P.R., Pfisterer M.E., Stauffer J.C., Erne P., Urban P., Investigators A.P.R. (2008). Ten-year trends in the incidence and treatment of cardiogenic shock. Ann. Intern. Med..

[7] Sjauw K.D., Engstrom A.E., Vis M.M., van der Schaaf R.J., Baan J., Koch K.T., de Winter R.J., Piek J.J., Tijssen J.G., Henriques J.P. (2009). A systematic review and meta-analysis of intra-aortic balloon pump therapy in ST-elevation myocardial infarction: Should we change the guidelines?. Eur. Heart J..

[8] Goldberg R.J., Spencer F.A., Gore J.M., Lessard D., Yarzebski J. (2009). Thirty-year trends (1975 to 2005) in the magnitude of, management of, and hospital death rates associated with cardiogenic shock in patients with acute myocardial infarction: A population-based perspective. Circulation.

[9] Hochman J.S., Buller C.E., Sleeper L.A., Boland J., Dzavik V., Sanborn T.A., Godfrey E., White H.D., Lim J., LeJemtel T. (2000). Cardiogenic shock complicating acute myocardial infarction—Etiologies, management and outcome: A report from the SHOCK Trial Registry. SHould we emergently revascularize Occluded Coronaries for cardiogenic shocK?. J. Am. Coll. Cardiol..

[10] Lauridsen M.D., Rorth R., Lindholm M.G., Kjaergaard J., Schmidt M., Moller J.E., Hassager C., Torp-Pedersen C., Gislason G., Kober L. (2020). Trends in first-time hospitalization, management, and short-term mortality in acute myocardial infarction-related cardiogenic shock from 2005 to 2017: A nationwide cohort study. Am. Heart J..

[11] Hochman J.S., Sleeper L.A., Webb J.G., Sanborn T.A., White H.D., Talley J.D., Buller C.E., Jacobs A.K., Slater J.N., Col J. (1999). Early revascularization in acute myocardial infarction complicated by cardiogenic shock. SHOCK Investigators. Should We Emergently Revascularize Occluded Coronaries for Cardiogenic Shock. N. Engl. J. Med..

[12] Thiele H., Zeymer U., Neumann F.J., Ferenc M., Olbrich H.G., Hausleiter J., de Waha A., Richardt G., Hennersdorf M., Empen K. (2013). Intra-aortic balloon counterpulsation in acute myocardial infarction complicated by cardiogenic shock (IABP-SHOCK II): Final 12 month results of a randomised, open-label trial. Lancet.

[13] Unverzagt S., Buerke M., de Waha A., Haerting J., Pietzner D., Seyfarth M., Thiele H., Werdan K., Zeymer U., Prondzinsky R. (2015). Intra-aortic balloon pump counterpulsation (IABP) for myocardial infarction complicated by cardiogenic shock. Cochrane Database Syst. Rev..

[14] Neumann F.J., Sousa-Uva M., Ahlsson A., Alfonso F., Banning A.P., Benedetto U., Byrne R.A., Collet J.P., Falk V., Head S.J. (2019). 2018 ESC/EACTS Guidelines on myocardial revascularization. Eur. Heart J..

[15] Schrage B., Becher P.M., Bernhardt A., Bezerra H., Blankenberg S., Brunner S., Colson P., Cudemus Deseda G., Dabboura S., Eckner D. (2020). Left Ventricular Unloading Is Associated With Lower Mortality in Patients With Cardiogenic Shock Treated With Venoarterial Extracorporeal Membrane Oxygenation: Results From an International, Multicenter Cohort Study. Circulation.

[16] Smith M., Vukomanovic A., Brodie D., Thiagarajan R., Rycus P., Buscher H. (2017). Duration of veno-arterial extracorporeal life support (VA ECMO) and outcome: An analysis of the Extracorporeal Life Support Organization (ELSO) registry. Crit. Care.

[17] Seyfarth M., Sibbing D., Bauer I., Frohlich G., Bott-Flugel L., Byrne R., Dirschinger J., Kastrati A., Schomig A. (2008). A randomized clinical trial to evaluate the safety and efficacy of a percutaneous left ventricular assist device versus intra-aortic balloon pumping for treatment of cardiogenic shock caused by myocardial infarction. J. Am. Coll. Cardiol..

[18] Ouweneel D.M., Eriksen E., Seyfarth M., Henriques J.P. (2017). Percutaneous Mechanical Circulatory Support Versus Intra-Aortic Balloon Pump for Treating Cardiogenic Shock: Meta-Analysis. J. Am. Coll. Cardiol..

[19] Tsao N.W., Shih C.M., Yeh J.S., Kao Y.T., Hsieh M.H., Ou K.L., Chen J.W., Shyu K.G., Weng Z.C., Chang N.C. (2012). Extracorporeal membrane oxygenation-assisted primary percutaneous coronary intervention may improve survival of patients with acute myocardial infarction complicated by profound cardiogenic shock. J. Crit. Care.

[20] Schrage B., Ibrahim K., Loehn T., Werner N., Sinning J.M., Pappalardo F., Pieri M., Skurk C., Lauten A., Landmesser U. (2019). Impella Support for Acute Myocardial Infarction Complicated by Cardiogenic Shock. Circulation.

[21] Windecker S., Kolh P., Alfonso F., Collet J.P., Cremer J., Falk V., Filippatos G., Hamm C., Head S.J., Authors/Task Force Members (2014). 2014 ESC/EACTS Guidelines on myocardial revascularization: The Task Force on Myocardial Revascularization of the European Society of Cardiology (ESC) and the European Association for Cardio-Thoracic Surgery (EACTS)Developed with the special contribution of the European Association of Percutaneous Cardiovascular Interventions (EAPCI). Eur. Heart J..

[22] Mehta L.S., Beckie T.M., DeVon H.A., Grines C.L., Krumholz H.M., Johnson M.N., Lindley K.J., Vaccarino V., Wang T.Y., Watson K.E. (2016). Acute Myocardial Infarction in Women: A Scientific Statement From the American Heart Association. Circulation.

[23] Cenko E., Yoon J., Kedev S., Stankovic G., Vasiljevic Z., Krljanac G., Kalpak O., Ricci B., Milicic D., Manfrini O. (2018). Sex Differences in Outcomes After STEMI: Effect Modification by Treatment Strategy and Age. JAMA Intern. Med..

[24] Kuehnemund L., Koeppe J., Feld J., Wiederhold A., Illner J., Makowski L., Gerss J., Reinecke H., Freisinger E. (2021). Gender differences in acute myocardial infarction-A nationwide German real-life analysis from 2014 to 2017. Clin. Cardiol..

[25] Lee J.H., Park J.H., Jang J.H., Kim S.H., Hong S.Y., Heo W., Lee D.H., Choi H.S., Kim K.H., Jang H.J. (2022). The role of nafamostat mesilate as a regional anticoagulant during extracorporeal membrane oxygenation. Acute Crit. Care.

[26] Li D.H., Sun M.W., Zhang J.C., Zhang C., Deng L., Jiang H. (2022). Is bivalirudin an alternative anticoagulant for extracorporeal membrane oxygenation (ECMO) patients? A systematic review and meta-analysis. Thromb. Res..

[27] Sanfilippo F., La Via L., Murabito P., Pappalardo F., Astuto M. (2022). More evidence available for the use of Bivalirudin in patients supported by extracorporeal membrane oxygenation. Thromb. Res..

[28] Sheu J.J., Tsai T.H., Lee F.Y., Fang H.Y., Sun C.K., Leu S., Yang C.H., Chen S.M., Hang C.L., Hsieh Y.K. (2010). Early extracorporeal membrane oxygenator-assisted primary percutaneous coronary intervention improved 30-day clinical outcomes in patients with ST-segment elevation myocardial infarction complicated with profound cardiogenic shock. Crit. Care Med..

[29] Knaus W.A., Draper E.A., Wagner D.P., Zimmerman J.E. (1985). APACHE II: A severity of disease classification system. Crit. Care Med..

