# Supplementary material for: Complications and Outcomes in 39,864 Patients Receiving Standard Care Plus Mechanical Circulatory Support or Standard Care Alone for Infarct-Associated Cardiogenic Shock
Source: J Clin Med. 2024 Feb 19;13(4):1167. doi: 10.3390/jcm13041167 (PMC10889198; doi:10.3390/jcm13041167)
Supplement: Supplementary file 1 [file jcm-13-01167-s001.zip › jcm-2870213-supplementary.pdf]

Supplemental Table S1: ICD-10-GM Codes/ OPS diagnosis and procedure codes for data retrieval

**International classification of disease 10th Revision, German Modification (ICD-10 GM)**	**Code**
Acute myocardial infarction (AMI)	I21.-; I22.-
Previous myocardial infarction (MI)	I21.-; I22.-
Cerebrovascular Disease (CVD)	I65.-; I66.-; I67.2
Previous stroke	I63.-; I64.-; I69.3; I69.4
Hypertension	I10.-; I11.-; I12.-; I13.-; 15.-
Diabetes mellitus (DM)	E10.-; E11.-; E12.-; E13.-; E14.-
Dyslipidemia	E78.-
Obesity	E66.-
Smoking	F17.-
Chronic heart failure (CHF)	I50.-
Chronic kidney disease (CKD)	N18.- ; N19.-
Diseased Coronary Vessels: 1	I25.11 (and no I25.12 or I25.13)
Diseased Coronary Vessels: 2	I25.12 (and no I25.13)
Diseased Coronary Vessels: 3	I25.13
Shock	T81.1; R57.0
Acute stroke	I63.-; I64.-
Hemorrhagic stroke	I60.-; I61.-; I62.-
Bleeding	K92.-; H44.8; T81.0; T81.2; T81.3; T81.7
Sepsis	A41;
Acute Renal Failure (ARF)	N17;
Previous (coronary artery bypass grafting) CABG	Z95.1 (with additional OPS codes)
Previous heart Valve implantation	Z95.2 (with additional OPS codes)
**German procedure classification system (OPS)**	
Blood transfusion	8-800.0; 8-800.1; 8-800.c
Percutaneous coronary intervention (PCI) (previous or current)	8-837;
Current CABG	5-36 (with additional ICD codes)

Current valve implantation	5-351; 5-352; 5-353; 5-354 (with additional ICD codes)
In-hospital-resuscitation	8-771
IABP	8-83a.0
Impella	8-83a.3
Veno-arterial extracorporeal membrane oxygenation (V-A ECMO)	8-852.3

Supplemental Table S2: Baseline characteristics (pooled)

	no tMCS (n=32,804)	tMCS (n=7,060)	P-value
Male, n (%)	19,615 (59.8)	4,855 (68.8)	<0.001
No of diseased coronary vessels: 0, n (%)	7,308 (22.3)	396 (5.61)	<0.001
No of diseased coronary vessels: 1, n (%)	4,056 (12.4)	744 (10.5)	<0.001
No of diseased coronary vessels: 2, n (%)	5,922 (18.1)	1,228 (17.4)	0.19
No of diseased coronary vessels: 3, n (%)	15,518 (47.3)	4,692 (66.5)	<0.001
Arterial hypertension, n (%)	28,907 (88.1)	6,009 (85.1)	<0.001
Diabetes, n (%)	17,073 (52.1)	3,650 (51.7)	0.60
Dyslipidemia, n (%)	21,893 (66.8)	4,954 (70.2)	<0.001
Obesitas, n (%)	8,850 (27.0)	2,061 (29.2)	<0.001
Smoking, n (%)	6,522 (19.9)	1,685 (23.9)	<0.001
Previous myocardial infarction, n (%)	9,653 (29.4)	2,396 (33.9)	<0.001
Previous stroke, n (%)	5,097 (15.5)	832 (11.8)	<0.001
Previous PCI, n (%)	2,356 (7.18)	593 (8.40)	<0.001
Previous CABG, n (%)	2,728 (8.32)	526 (7.45)	0.02
Previous valve replacement, n (%)	429 (1.31)	53 (0.75)	<0.001
Chronic heart failure, n (%)	23,244 (70.9)	5,463 (77.4)	<0.001
Chronic kidney disease, n (%)	14,090 (43.0)	2,788 (39.5)	<0.001
Chronic kidney disease with dialysis, n (%)	6,340 (19.3)	1,123 (15.9)	<0.001

Abbreviations: tMCS – temporary mechanical circulatory support; IABP - intra-aortic balloon pump; V-A ECMO – veno-arterial extracorporeal membrane oxygenation; IQR – interquartile range; PCI – percutaneous coronary intervention; CABG – coronary artery bypass graft

Supplemental Table S3: Cox regression

	Hazard ratio (95% CI)	P-Value
Female gender	1.02 (1.00-1.05)	0.079
IABP	0.89 (0.86-0.92)	<0.001
Impella	1.25 (1.15-1.35)	<0.001
V-A ECMO	1.57 (1.45-1.69)	<0.001
Age	1.04 (1.04-1.04)	<0.001
Arterial hypertension	0.83 (0.80-0.86)	<0.001
Diabetes	1.22 (1.19-1.25)	<0.001
Dyslipidemia	0.73 (0.71-0.75)	<0.001
Obesitas	1.03 (1.00-1.06)	0.036
Smoking	0.92 (0.89-0.95)	<0.001
Previous myocardial infarction	0.73 (0.71-0.75)	<0.001
Previous stroke	1.22 (1.18-1.26)	<0.001
Previous PCI	1.27 (1.21-1.32)	<0.001
Previous CABG surgery	1.19 (1.14-1.24)	<0.001
Previous valve replacement	1.28 (1.16-1.42)	<0.001
Chronic heart failure	0.71 (0.69-0.73)	<0.001
Chronic kidney disease	0.98 (0.95-1.00)	0.087

Abbreviations: CI - confidence interval; IABP - intra-aortic balloon pump; V-A ECMO – veno-arterial extracorporeal membrane oxygenation; PCI – percutaneous coronary intervention; CABG – coronary artery bypass graft
